# Effectiveness of diet modification on dietary nutrient intake, aspiration, and fluid intake for adults with dysphagia: a meta-analysis of randomized controlled trials

**DOI:** 10.1016/j.jnha.2025.100486

**Published:** 2025-01-15

**Authors:** Yu-Hao Chu, Jane C-J Chao

**Affiliations:** a School of Nutrition and Health Sciences, Taipei Medical University, Taipei, Taiwan; b TMU Research Center for Digestive Medicine, Taipei Medical University, Taipei, Taiwan; c Nutrition Research Center, Taipei Medical University Hospital, Taipei, Taiwan

**Keywords:** Dysphagia, Thickened fluid, Texture-modified diet, Dietary nutrient intake, Fluid intake, Aspiration

## Abstract

**Objectives:**

To determine and explore the effectiveness of diet modification on dietary nutrient intake, aspiration, and fluid intake in adults with dysphagia.

**Participants:**

Adults with dysphagia.

**Design:**

A meta-analysis of randomized controlled trials (RCTs).

**Methods:**

We conducted a comprehensive literature search in EMBASE, Cochrane Library, Ovid-Medline, CINAHL, Web of Science, PubMed, and reference lists until November 2023. Quality of the included studies was assessed by the Cochrane Risk of Bias Assessment tool 2.0. Data analysis was performed using Comprehensive Meta-analysis 3.0 for pooled Hedges’ *g* and odds ratios (ORs) with corresponding confidence interval (CI) adopting a random-effects model. A X^2^- based test using Cochran’s Q (*P* < 0.10) and $I^{2}$ statistics evaluated heterogeneity.

**Results:**

In total, 16 RCTs from among 3,854 studies retrieved from the literature search with 1812 adults with dysphagia were included in this meta-analysis. Texture-modified diets revealed a significant small effect on increasing energy intake (*g*: 0.37, 95% CI = 0.05–0.68) and a medium effect on increasing protein intake (*g*: 0.56, 95% CI = 0.13–0.99). Thickened fluids revealed a significantly reduced risk of aspiration (OR: 0.59, 95% CI = 0.44–0.79), and thickened fluids combined with water protocol revealed a significant large effect on increasing fluid intake (*g*: 3.96, 95% C = 0.75–7.16).

**Conclusions:**

The findings of this meta-analysis demonstrated that texture-modified diets increase dietary intake of energy and protein for adults with dysphagia. In addition, thickened fluids reduced the risk of aspiration while thickened fluids combined with water protocol increased fluid intake in adults with dysphagia.

## 1 Introduction

Swallowing is a complex sensorimotor process regulated by the central pattern generator in the medulla oblongata and involves a neural network controlling 25 paired muscles of the oral cavity, pharynx, larynx, and esophagus [1]. It comprises four phases: oral-preparatory, oral-transit, pharyngeal, and esophageal [2]. Disruption of any of these phases due to poor coordination or damage to the oropharyngeal and esophageal structures and neural network lead to dysphagia [2,3]. The global prevalence of dysphagia in adults was estimated at 60%, considering different baseline conditions [4]. Dysphagia due to neurogenic causes is prevalent in 44.8% of multiple sclerosis cases [5], 42% of acute stroke cases [6], 36.9% of Parkinson’s disease cases [7], and 35% of hospitalized coronavirus disease 2019 patients [8]. Structural causes affect 46% of older adults [9] and 45.3% of head and neck cancer patients [10]. Dysphagia is highly associated with pulmonary complications including aspiration pneumonia, malnutrition, weight loss, dehydration, and mortality [2,6,9,11]. Therefore, early identification, prevention, and management of dysphagia are crucial to preventing complications, improving quality of life, and reducing mortality in adults with dysphagia.

Diet modification, including texture-modified diets (TMDs) and thickened fluids (TFs), is crucial for dysphagia management to ensure the safe transport of solid and liquid boluses and prevent aspiration and penetration, thereby protecting the airway [2,3,12]. TMDs and TFs involve altering the texture, viscosity, and thickness of foods and fluids to meet the nutritional needs of adults with dysphagia [[13], [14], [15], [16]]. Several frameworks have been developed globally to standardize these diets, including (1) the International Dysphagia Diet Standardisation Initiative [15,17], (2) National Dysphagia Diet [18], (3) Australian Fluid Texture Modification Scale [13], and (4) Japanese Dysphagia Diet 2021 [19]. Moreover, these frameworks provide a comprehensive and standardized approach to dysphagia management, ensuring consistency, safety, and efficacy for adults with dysphagia across various cultural and healthcare settings.

A systematic review by Hansen et al. (2022) found no convincing evidence that TMDs and TFs prevented death or pneumonia, or improved oral intake, the nutritional status, or quality of life in individuals with oropharyngeal dysphagia [20]. Kaneoka et al. in 2017 also reported no difference between TFs and thin fluids in preventing pneumonia risk in adults with dysphagia [21]. Conversely, Wu et al. in 2020 showed that shaped TMDs effectively improved dietary energy and protein intake levels in older adults [22]. However, (1) evidence of the effectiveness of diet modifications (TMDs/TFs) on dietary nutrient intake (energy, protein, fat, carbohydrate, sodium, and fiber), aspiration, fluid intake, and body composition (body mass index (BMI) and body weight (BW)) in adults with dysphagia remains unclear and debatable and (2) limited evidence exists on the overall effectiveness of diet modifications (TMDs/TFs) [[20], [21], [22]]. The current study was conducted to answer the research question on what the efficacy of modified-texture diet and thickened fluids on dietary nutrient intake, aspiration, fluid intake, and body composition in people with dysphagia is. Therefore, to address these knowledge gaps and answer the main research question, we conducted a meta-analysis of randomized controlled trials (RCTs) to determine the effectiveness of diet modifications (TMDs/TFs) on dietary nutrient intake (energy, proteins, fats, fiber, carbohydrates, and sodium), aspiration, fluid intake, and body composition (BMI and BW) in adults with dysphagia.

## 2 Methods

### 2.1 Search strategy

This meta-analysis followed the reporting guidelines in the updated 2020 Preferred Reporting Items for Systematic Reviews and Meta-Analyses (PRISMA) statement [23]. The study protocol was registered in the Prospective Register of Systematic Review (PROSPERO) (ID: CRD42023450638). A comprehensive literature search was conducted in PubMed, CINAHL, Ovid-MEDLINE, Cochrane Library, and Web of Science from the inception of each database until November 2023. The database search was conducted using the following keywords in combination: elderly, aged, old age, adults, older adults, or old people, and modified diet or texture-modified diets, puree diet, pureed diet, mince diet, blend diet, chop diet, soft diet, soften diet, thicken diet, liquid diet, liquefied diet, thickened diet, fluid thickeners, thickened fluids, liquid thickeners, thickened liquids, gum-based thickeners, modified starch thickeners, or starch thickeners, and dysphagia, swallowing disorder, deglutition disorder, or oropharyngeal dysphagia (Supplementary Table S1). Additionally, reference lists of previous relevant systematic reviews, meta-analyses, and randomized controlled studies were also searched and reviewed to identify other potential studies for inclusion, and then a Google search was performed to identify other potential studies for inclusion. Corresponding authors of previously published studies were also contacted through email to provide additional data to ensure that all potential eligible studies were found and included. The study followed the design of the registered study protocol, and did not deviate from the registered protocol.

### 2.2 Study selection – inclusion and exclusion criteria

**Fig. 1:**
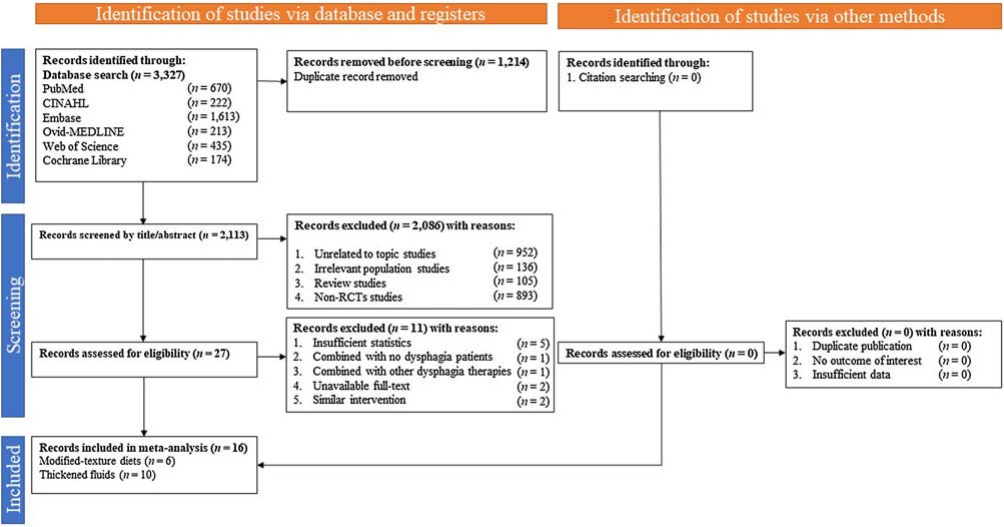
PRISMA flowchart for study selection.

The screening of the studies was performed manually using Endnote version 20.1 software by two independent reviewers (Y.H.C. and J.K.B). For consistency, the findings of the two reviewers were compared, and the selection of the included studies in the meta-analysis was based on the population, intervention or exposure, comparison, outcomes, and study design criteria. The study selection of included studies was based on the population, intervention or exposure, comparison, outcomes, and study design criteria. The study inclusion criteria of the current meta-analysis were as follows: (1) the population included adults aged ≥18 years with dysphagia, (2) the intervention included TMDs or TFs, (3) the comparison included an active or passive control including nutritional advice or traditional diet or thin fluids, and (4) the study design was an RCT exclusively to ensure methodological consistency and minimize bias generating comparable results. Exclusion criteria of the current meta-analysis included duplicate studies, studies unrelated to this topic, non-relevant population studies, study protocols, systematic review or meta-analysis studies, and non-randomized studies (Fig. 1).

### 2.3 Data extraction and study outcomes

Data of the included studies were extracted by two independent reviewers (Y.H.C. and K.J.B.) using pre-specified data extraction forms designed by the authors. The extracted data included the first author last name and year of publication; mean age of participants, gender, cause of dysphagia, sample size, intervention type (TMDs, TFs, and TFs combined with water protocol), outcome indicators such as dietary nutrient intake (energy, protein, fat, carbohydrate, sodium, and fiber), aspiration, fluid intake, and body composition (BMI and BW), and outcome assessment time points (baseline and post-intervention). Primary outcomes were dietary nutrient intake (energy, protein, fat, carbohydrate, sodium, and fiber) and aspiration. Secondary outcomes were fluid intake and body composition (BMI and BW). Disagreements between reviewers (Y.H.C. and K.J.B.) were resolved by consensus through discussion with a third expert reviewer (J.C-J.C.).

### 2.4 Quality of the included studies

The Cochrane Handbook for Systematic Reviews of Interventions version 2.0 was used by two independent reviewers to assess the risk of bias of the included studies [24]. The Cochrane risk of bias assessment tool consists of five domains including bias due to missing outcome data, bias due to deviations from intended interventions, bias arising from the randomization process, bias in selection of the reported results, and bias in the measurement of the outcome. Each domain was rated as having a low risk of bias, some concerns of risk of bias, and a high risk of bias. The overall quality of each included study was rated by summing each criterion for the five risk of bias domains. Disagreements between reviewers were resolved by consensus through discussion with a third expert reviewer.

### 2.5 Statistical analysis–effect size calculation

Comprehensive Meta-Analysis, version 3.0 [25] was used to calculate Hedge’s g for dietary nutrient intake (energy, protein, fat, carbohydrate, sodium, and fiber), fluid intake, and body composition (BMI and BW) [26]. The pooled odds ratio (OR) for aspiration with corresponding confidence intervals (CIs) as pooled effect sizes were used to assess the effectiveness of TMDs and TFs between the experimental and control groups [26]. Means and standard deviations (SDs) were used to calculate the pooled effect size for continuous outcomes, while the number of participants with aspiration were compared between the experimental and control groups [27,28]. Furthermore, the Cochrane Handbook was used to calculate the SDs in studies that had a mean with the CI [29]. Moreover, Hozo et al. [30] and Wan et al. [31] were used to calculate the mean and SD using the sample size in the experimental and control groups in studies that presented the median and interquartile range (IQR). A random-effects model was adopted in the final analysis taking into account variations among the included studies [32]. A X^2^- based test presenting Cochrane’s Q statistic with statistically significant results that indicated variations among the included studies in the effectiveness of TMDs and TFs in adults with dysphagia was used to assess heterogeneity (*P* < 0.10) [33,34]. In addition, the $I^{2}$ statistic was used to quantify heterogeneity with the cutoff values of 25%, 50%, and 75%, respectively, indicating low, moderate, and high heterogeneity. Publication bias was examined by Egger’s regression test [35] and was considered significant at *P* < 0.05 [36].

The pooled effect size (Hedge’s *g*) for dietary nutrient intake (energy, protein, fat, carbohydrate, sodium, and fiber), fluid intake, and body composition (BMI and BW) was interpretated as a small effect at <0.2 to <0.49, as a medium effect at ≥0.5 to <0.79, and as a large effect at ≥0.8 with *P* < 0.05 indicating statistical significance [37]. The pooled effect size (OR) for aspiration was interpreted as a small effect at 1.50, a medium effect at 2.50, and a large effect at 4.30 with *P* < 0.05 indicating statistical significance [38]. A sensitivity analysis was performed by excluding studies with a high risk of bias in continuous outcomes (dietary nutrient intake (energy, protein, fat, carbohydrate, sodium, and fiber), fluid intake, and body composition (BMI and BW) and binary outcomes (aspiration) to check the robustness of the study findings.

### 2.6 Moderator analysis

In the presence of statistically significant moderate and high heterogeneity on the effectiveness of TMDs and TFs on dietary nutrient intake (energy, protein, fat, carbohydrate, sodium, and fiber), fluid intake, aspiration, and body composition (BMI and BW) in adults with dysphagia, key study variables were used to identify potential significant moderator variables [39,40]. Subgroup analyses of categorical variables included gender (male or female), etiology of dysphagia, type of TMDs, type of TFs, type of control group, study quality, and study design. A meta-regression analysis was performed for age. Subgroup analyses were limited to groups with at least two studies for sufficient data among the included studies.

## 3 Results

### 3.1 Search results

In total, 3,327 studies were retrieved from the databases of PubMed (*n* = 670), CINAHL (*n* = 222), Embase (*n* = 1,613), Ovid-MEDLINE (*n* = 213), Web of Science (*n* = 435), and Cochrane library (*n* = 174), and reference lists of previously published systematic reviews and meta-analyses (*n* = 0) (Fig. 1**)**. In total, 1,214 study duplicates were removed from the identified 3,327 studies, leaving 2,113 studies that were screened by title and abstracts. Twenty-seven eligible studies were identified after 2,113 studies were excluded based on being unrelated to the topic (*n* = 952), having an irrelevant population (*n* = 136), being a review or meta-analysis (*n* = 105), and not being an RCT (*n* = 893). Finally, 16 full-text studies [[41], [42], [43], [44], [45], [46], [47], [48], [49], [50], [51], [52], [53], [54], [55], [56]] published between 1997 and 2022 met the inclusion criteria after eleven studies were excluded based on being combined with no dysphagia patients (*n* = 1), combined with other dysphagia therapies (*n* = 1), insufficient outcomes for analysis (*n* = 5), unavailable full-texts (*n* = 2), and having similar interventions (*n* = 2) (Supplementary Table S3). Among these 16 included studies, six studies focused on TMDs, and ten studies focused on TFs.

### 3.2 Study characteristics

Overall, 1,812 participants were found in the included studies with sample sizes ranging 14–711 (Table 1). Regarding gender, 1,145 participants were men representing 63% and 667 were women representing 37% of the total identified participants. Participants were randomly assigned to an experimental (*n* = 471) and a control (*n* = 469) group, while three crossover randomized studies with 711, 100, and 61 participants used participants in both the experimental and control groups. Average ages in the experimental group ranged 62.0–85.6 years, whereas average ages in the control groups ranged 62.0–84.6 years. Among the 16 included studies, ten studies were parallel group RCTs, while six studies were crossover randomized trials. The primary diagnosis of participants was dysphagia in adults due to stroke, dementia, and Parkinson’s disease. Regarding the sources of funding, all the included studies received financial support with no conflict of interest. The included studies were conducted in geriatric institutions, nursing homes, and hospitals.

**Table 1:** Study and interventional characteristics on the effectiveness of texture-modified diets and thickened fluids.

Author (Year) design	Age (years) setting	Diagnosis/condition	Size gender	Intervention group	Control group	Study outcomes	Assessment duration
Côté et al., 2017 RCT	EG: 76.4 (11.8) CG: 80.3 (11.8) Nursing home	Mixed etiology for dysphagia	Total: 17 EG: 7 CG: 10 Male: 2 Female: 15	Texture- modified diet (Epikura foods)	Traditional diet	**Primary outcomes** Dietary nutrient intake Energy Protein Fat Carbohydrate Fiber Mean intake per day **Secondary outcomes** Fluid intake NA Body composition Body mass index NA Body weight	Baseline 24 weeks
Diniz et al., 2009 Crossover RCT	EG: 63.4 (13.4) CG: Hospital	Stroke	Total: 61 EG: 61 CG: 61 Male: 40 Female: 21	Thickened fluids (spoon thick diet)	Liquid diet	**Primary outcomes** Dietary nutrient intake NA Aspiration Nasoendoscopy **Secondary outcomes** Fluid intake NA Body composition NA	Baseline 1 week
Garon et al., 1997 Crossover RCT	EG: 77.5 CG: Rehabilitation unit	Stroke	Total: 20 EG: 10 CG: 10 Male: 14 Female: 6	Thickened fluids	Non-thickened fluids	**Primary outcomes** Dietary nutrient intake NA Aspiration NA **Secondary outcomes** Fluid intake Mean intake per day Body composition NA	Baseline 4 weeks
Germain et al., 2006 RCT	EG: 82.5 (4.4) CG: 84.6 (3.8) Long-term care facility	Mixed etiology for dysphagia	Total: 17 EG: 8 CG: 9 Male: 7 Female: 10	Texture- modified diet (pureed-texture foods with thickened beverages)	Traditional diet	**Primary outcomes** Dietary nutrient intake Energy Protein Fat Carbohydrate Sodium Fiber Mean intake per day Aspiration NA **Secondary outcomes** Fluid intake NA Body composition NA	Baseline 12 weeks
Goulding & Bakheit, 2000 RCT	EG: 75.0 (10.3) CG: 75.9 (8.3) Rehabilitation unit	Stroke	Total: 46 EG: 23 CG: 23 Male: 23 Female: 23	Thickened fluids (yogurt consistency fluids)	Thickened fluids (syrup consistency fluids)	**Primary outcomes** Dietary nutrient intake NA Aspiration Pulse oximetry **Secondary Outcomes** Fluid intake NA Body composition NA	Baseline 1 week
Higashiguchi et al., 2017 Crossover RCT	EG: 78.3 (11.2) CG: 75.5 (12.6) Hospital	Mixed etiology for dysphagia	Total: 50 EG: 27 CG: 23 Male: 27 Female: 23	Texture- modified diet (iEAT diet)	Traditional diet	**Primary outcomes** Dietary nutrient intake Energy Protein Fat Carbohydrate Sodium Fiber Mean intake per day Aspiration NA **Secondary outcomes** Fluid intake NA Body composition Body weight	Baseline 1 week
Karagiannis et al., 2011 RCT	EG: 79.0 (11.0) CG: 80.0 (7.0) Hospital	Mixed etiology	Total: 76 EG: 42 CG: 34 Male: 39 Female: 37	Thickened fluids	Non-thickened fluids	**Primary outcomes** Dietary nutrient intake NA Aspiration NA **Secondary outcomes** Fluid intake Mean intake per day Body composition NA	Baseline 2 weeks
Leonard et al., 2013 RCT	EG1: 62.0 (13.0) EG2: 62.0 (13.0) CG: 62.0 (13.0) Hospital	Mixed etiology	Total: 100 EG1: 100 EG2: 100 CG: 100 Male: 58 Female: 42	Thickened fluids (gum-based) Thickened fluids (starch-based)	Thin fluids	**Primary outcomes** Dietary nutrient intake NA Aspiration VFSS **Secondary outcomes** Fluid intake NA Body composition NA	Baseline 1 week
Logemann et al., 2008 Crossover RCT	EG1: 80 EG2: 80 CG: 80 Hospital	Dementia/Parkinson’s disease	Total: 711 EG1: 711 EG2: 711 CG: 711 Male: 498 Female: 213	Thickened fluids (nectar-thick fluids) Thickened fluids (honey-thick fluids)	Chin-down posture	**Primary outcomes** Dietary nutrient intake NA Aspiration VFS **Secondary outcomes** Fluid intake NA Body composition NA	Baseline 12 weeks
Murray et al., 2016 RCT	EG: 78.0 (6.8) CG: 80.0 (6.4) Hospital	Stroke	Total: 14 EG: 8 CG: 6 Male: 10 Female: 4	Thickened fluids	Non-thickened fluids	**Primary outcomes** Dietary nutrient intake NA Aspiration NA **Secondary outcomes** Fluid intake Mean intake per day Body composition NA	Baseline 2 weeks
Reyes-Torres et al., 2019 RCT	EG: 75.5 (9.9) CG: 76.0 (9.9) Hospital	Mixed etiology	Total: 40 EG: 20 CG: 20 Male: 20 Female: 20	Texture- modified diet (texture-modified foods and thickened fluids)	Traditional diet	**Primary outcomes** Dietary nutrient intake NA Aspiration NA **Secondary outcomes** Fluid intake NA Body composition Body mass index BIA Body weight	Baseline 12 weeks
Robbins et al., 2008 RCT	EG1: 80 EG2: 81 CG: 81 Hospital	Dementia/Parkinson’s disease	Total: 515 EG1: 133 EG2: 123 CG: 259 Male: 359 Female: 156	Thickened fluids (nectar-thick fluids) Thickened fluids (honey-thick fluids)	Chin-down posture	**Primary outcomes** Dietary nutrient intake NA Aspiration VFS **Secondary outcomes** Fluid intake NA Body composition NA	Baseline 12 weeks
Salas-Salvado et al., 2005 RCT	EG: 85.6 (6.6) CG: 83.9 (6.9) Nursing homes	Alzheimer’s disease	Total: 53 EG: 24 CG: 29 Male: 44 Female: 9	A whole formula diet based on lyophilized foods	Nutritional advice	**Primary outcomes** Dietary nutrient intake Energy Protein Carbohydrate Mean intake per day Aspiration NA **Secondary outcomes** Fluid intake NA Body composition NA	Baseline 2 weeks
Salle et al., 2021 Crossover RCT	EG: 74.3 (13.5) CG: 74.3 (13.5) Hospital	Mixed etiology	Total: 30 EG: 15 CG: 15 Male: 15 Female: 15	Thickened fluids (ready-to-use gelled water	Thickened fluids (starch-based)	**Primary outcomes** Dietary nutrient intake NA Aspiration PASS **Secondary outcomes** Fluid intake NA Body composition NA	Baseline 2 weeks
Vidal-Casariego et al., 2021 RCT	EG: 79.1 (12.4) CG: 79.4 (12.4) Hospital	Mixed etiology	Total: 40 EG: 20 CG: 20 Male: 20 Female: 20	Thickened fluids (gum-based)	Thickened fluids (starch-based)	**Primary outcomes** Dietary nutrient intake NA Aspiration NA **Secondary outcomes** Fluid intake Mean intake per day Body composition NA	Baseline 1 week
Wu et al., 2022 Crossover RCT	EG: 83.2 (7.3) CG: 83.2 (7.3) Hospital	Mixed etiology	Total: 22 EG: 11 CG: 11 Male: 10 Female: 12	Texture- modified diet (hydrolyzed meat)	Texture- modified diet (freshly made pureed meat)	**Primary outcomes** Dietary nutrient intake Energy Protein Fat Carbohydrate Sodium Fiber Mean intake per day Aspiration NA **Secondary outcomes** Fluid intake NA Body composition Body mass index BIA Body weight	Baseline 6 weeks

BIA, bioelectrical impedance analysis; CG, control group; EG, experimental group; NA, not available; PAS, Penetration Aspiration Scale; PASS, Practical Aspiration Screening Scheme test; RCT, randomized controlled trial; VFS, videofluoroscopy; VFSS, Videofluoroscopic Swallowing Study.

### 3.3 Interventional characteristics

Two types of interventions were included in the current study: TMDs and TFs. The experimental groups for TMDs received Epikura foods, reshaped minced-, or pureed-texture foods with thickened beverages, the iEAT diet, and texture-modified foods and thickened drinks diet, with nectar or pudding viscosity and a controlled bolus volume. The experimental groups with TFs received a spoon thick diet, thickened liquids, yogurt consistency liquids, gum-based thickened liquids, nectar-thick liquids, honey-thick fluids, and ready-to-use gelled water. The active and passive control groups for the TMDs received institutional texture-modified diets, traditional nourishment, modified traditional diets, nutritional advice, and isocaloric standard treatment. The active and passive control groups for the TFs were liquid diets, syrup consistency fluids, thin fluids, chin-down posture, and non-TFs. Study outcomes included dietary nutrient intake (energy, protein, fat, carbohydrate, sodium, and fiber), aspiration, fluid intake, and body composition (BMI and BW). Study periods of the included studies ranged 1–24 weeks (Table 1).

### 3.4 Quality of the included studies

The current meta-analysis included six crossover and ten parallel-design randomized controlled studies that were assessed with the Cochrane’s risk of bias assessment tool. Regarding crossover design randomized controlled studies, three studies had a low risk of bias indicative of being of high quality, while three studies had some concerns in the risk of bias indicative of being of moderate quality (Supplementary Fig. S1). Regarding parallel design randomized controlled studies, three studies had a low risk of bias indicative of being high quality studies, five studies had some concerns in the risk of bias indicative of being moderate quality studies, and two studies had high risk of bias indicative of being poor quality studies (Supplementary Fig. S2). Overall, five studies were of high quality, nine studies were of moderate quality, and two studies were of poor quality.

### 3.5 Effectiveness of TMDs on energy and protein intake

**Fig. 2:**
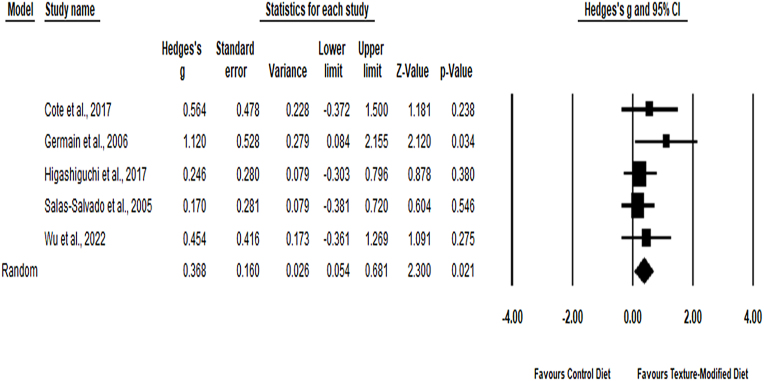
Effectiveness of texture-modified diets on energy intake.

Five studies were used to explore the effectiveness of TMDs on energy intake. The meta-analysis revealed a small to medium effect on improving energy intake (Hedge’s *g*: 0.37, 95% CI = 0.05–0.68, *P* < 0.02) with low heterogeneity (Q-statistic: 2.92, ${\text{I}}^{2\;}$ = 0%, *P* = 0.57) (Table 2, Fig. 2). The findings suggested that TMDs increased and improved energy intake. Some evidence of publication bias with Egger’s regression intercept of 2.73 and a *P* value of 0.03 was observed.

**Table 2:** Effectiveness of texture-modified diets/thickened fluids (random-effect model).

Study outcomes	*n*	Effect size			Heterogeneity			
Primary outcomes		Hedge’s *g*/OR (95% CI)	Z-value	*P* value	Q-statistic	$I^{2}$	Tau^2^	*P* value
Energy (*g*)	4	0.37 (0.05–0.68)*	2.30	0.02	2.92	0%	0.00	0.57
Protein (*g*)	4	0.56 (0.13–0.99)*	2.55	0.01	3.59	16.6%	0.13	0.31
Fat (*g*)	4	0.46 (−0.06–0.98)	1.74	0.08	4.93	39.1%	0.11	0.18
Carbohydrate (*g*)	4	−0.01 (−0.39–0.36)	−0.53	0.95	1.49	0%	0.00	0.69
Sodium (*g*)	3	0.26 (−0.15–0.67)	1.24	0.22	0.33	0%	0.00	0.85
Fiber (*g*)	2	−0.36 (−1.13–0.59)	−0.75	0.45	1.97	48.8%	0.89	0.16
Aspiration (OR)	6	0.59 (0.44–0.79)*	−3.56	<0.001	21.5	62.9%	0.09	<0.006
Secondary outcomes								
Fluid intake (*g*)	4	3.96 (0.75–7.16)*	2.13	0.02	136.00	97.8%	0.00	<0.0001
Body mass index (*g*)	2	0.20 (−0.29–0.68)	0.79	0.43	0.03	0%	0.43	0.85
Body weight (*g*)	4	0.18 (−0.16–0.52)	1.05	0.29	0.83	0%	0.00	0.84

CI, confidence interval; *n*, number of studies; OR, odds ratio.

* Significant pooled effect estimate.

Four studies were used to investigate the effectiveness of TMDs on protein intake. The meta-analysis revealed a medium effect on improving protein intake (Hedge’s *g*: 0.56, 95% CI = 0.13–0.99, *P* < 0.01) with low heterogeneity (Q-statistic: 3.59, $I^{2}$ = 16.6%, *P* = 0.31) (Table 2, Supplementary Fig. S3). The findings suggested that TMDs had a significant effect on increasing and improving protein intake. No evidence of publication bias with Egger’s regression intercept of −0.41 and a *P* value of 0.89 was found.

### 3.6 Effectiveness of TMDs on fat, carbohydrate, sodium, and fiber intake

Four studies explored the effectiveness of TMDs on fat intake. The meta-analysis revealed a non-significant large effect on increasing fat intake (Hedge’s *g*: 0.46, 95% CI = −0.06–0.98, *P* = 0.08) with low heterogeneity (Q-statistic: 4.93, $I^{2}$ = 39.1%, *P* = 0.12) (Table 2, Supplementary Fig. S4).

Four studies determined the effectiveness of TMDs on carbohydrate intake. The meta-analysis revealed a non-significant small effect on improving carbohydrate intake (Hedge’s *g*: −0.01, 95% CI = −0.39–0.36, *P* = 0.95) with low heterogeneity (Q-statistic: 1.49, $I^{2\;}$ = 0%, *P* = 0.69) (Table 2, Supplementary Fig. S5).

Three studies investigated the effectiveness of TMDs on sodium intake. The meta-analysis revealed a non-significant small effect on improving sodium intake (Hedge’s *g*: 0.26, 95% CI = −0.15–0.67, *P* = 0.22) with low heterogeneity (Q-statistic: 0.33, $I^{2}$ = 0%, *P* = 0.85) (Table 2, Supplementary Fig. S6). The findings suggested that TMD had a non-significant effect on increasing fat, fiber, carbohydrate, and sodium intake.

Two studies explored the effectiveness of TMDs on dietary fiber intake. The meta-analysis revealed a non-significant effect on improving fiber intake (Hedge’s *g*: −0.36, 95% CI = −1.13–0.59, *P* = 0.45) with low heterogeneity (Q-statistic: 1.95, ${\text{I}}^{2}$ = 48.8%, *P* = 0.16) (Table 2, Supplementary Fig. S7).

### 3.7 Effectiveness of TFs on aspiration

**Fig. 3:**
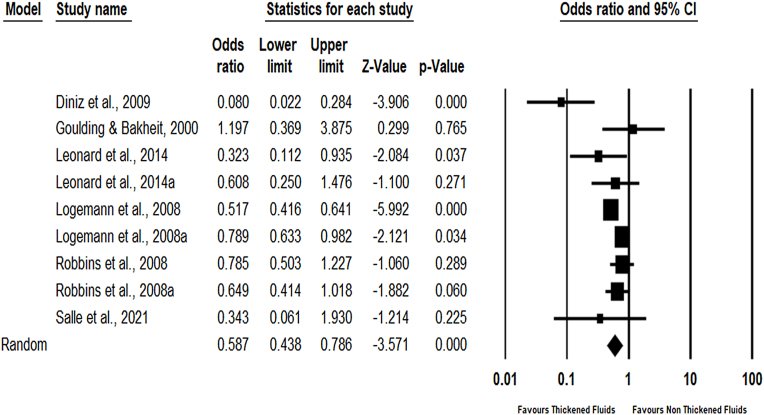
Effectiveness of thickened fluids on aspiration.

Six studies with nine effect estimates analyzed the effectiveness of TFs on the risk of aspiration. The meta-analysis revealed that TFs were associated with a lower risk of aspiration (OR: 0.59, 95% CI = 0.44–0.79, *P* < 0.001) with moderate heterogeneity (Q-statistic = 21.5, $I^{2}$ = 62.9%, *P* < 0.006) (Table 2, Fig. 3). The findings revealed that TFs were associated with a significant 41% reduction in the risk of aspiration in the experimental group compared to the control group. There was no evidence of publication bias with Egger’s regression intercept of −0.87 and a *P* value of 0.37.

Regarding the moderator analysis, age (OR: 0.04, 95% CI = 0.002–0.08, *P* < 0.04) was demonstrated to be a significant moderator variable for the effectiveness of TFs on aspiration (Supplementary Table S2). The findings showed that with an increasing age, there was a decrease in the effectiveness of TFs on reducing aspiration. However, the sample size, female gender, type of disease, and type of thickener were not significant moderator variables for the effectiveness of TFs on aspiration. Regarding the sample size (*P* = 0.39), the effectiveness of TFs on reducing aspiration was not affected by the sample size (OR: 0.001, 95% CI = −0.001–0.002). Regarding gender (*P* = 0.63), the effectiveness of TFs on reducing aspiration was not affected by being female (OR: 0.01, 95% CI = −0.03–0.05). Regarding the etiology of dysphagia (*P* = 0.61), TFs reduced aspiration in stroke (OR: 0.31, 95% CI = 0.02–4.44), mixed etiology (OR: 0.59, 95% CI = 0.43–0.81), and PD patients (OR: 0.72, 95% CI = 0.52–0.98), but the differences were not significant. Regarding the type of thickener (*P* = 0.56), gum-based thickened fluids (OR: 0.63, 95% CI = 0.47–0.84) and starch-based thickened fluids (OR: 0.48, 95% CI = 0.20–1.12) were associated with reduced aspiration but the differences were not significant.

### 3.8 Effectiveness of TFs and a water protocol on fluid intake

Four studies investigated the effectiveness of TFs and a water protocol on fluid intake. The meta-analysis revealed a significant large effect on improving fluid intake (Hedge’s *g*: 3.96, 95% CI = 0.75–7.16, *P* < 0.02) with high heterogeneity (Q-statistic: 136.0, $I^{2}$ = 97.8%, *P* < 0.001) (Table 2, Supplementary Fig. S8). The findings demonstrated that TFs and a water protocol significantly increased fluid intake. There was no evidence of publication bias with Egger’s regression intercept of 13.7 and a *P* value of 0.12.

### 3.9 Effectiveness of TMDs on BMI and BW

Two studies explored the effect of TMDs on BMI. The findings revealed that TMDs had a non-significant small effect on BMI (Hedge’s *g*: 0.20, 95% CI = −0.29–0.68, *P* = 0.43) with low heterogeneity (Q-statistic: 0.03, $I^{2\;}$ = 0%, *P* = 0.85) (Table 2, Supplementary Fig. S9). The findings suggested that TMDs had a non-significant effect on improving BMI.

Four studies determined the effect of TMDs on BW. The findings revealed that TMDs had a non-significant small effect on BW (Hedge’s *g*: 0.18, 95% CI = −0.16–0.52, *P* = 0.29) with low heterogeneity (Q-statistic: 0.83, $I^{2}$ = 0%, *P* = 0.84) (Table 2, Supplementary Fig. S10). The findings suggested that TMDs had a non-significant effect on improving BW.

## 4 Discussion

The present meta-analysis of RCTs evaluated the effectiveness of TMDs and TFs on dietary nutrients intake (energy, protein, fat, carbohydrate, sodium, and fiber), aspiration, fluid intake, and body composition (BMI and BW). The study findings offer more comprehensive and extensive evidence regarding the effectiveness of TMDs and TFs in adults with dysphagia. The current meta-analysis showed that TMDs significantly increased the intake of dietary energy and protein. In addition, TFs were significantly associated with a reduced risk of aspiration, while TFs and a water protocol increased fluid intake. However, TMDs demonstrated no difference in dietary intake of fat, carbohydrate, sodium, or fiber or body composition including BMI and BW compared to the control diet.

### 4.1 TMDs and dietary nutrient intake

The current meta-analysis revealed that TMDs effectively increased dietary energy and protein intake in adults with dysphagia. These findings align with the study of Wu et al. [22], and the study reported significant increases in dietary energy and protein intake among the adults with dysphagia. Additionally, a prior systematic review highlighted that the rheological, sensory, and physical properties of TMDs were crucial for safe swallowing and effective bolus propulsion through the four phases of swallowing [57]. In contrast, Hansen et al. [20] revealed that thickened liquid or texture modified diet did not prevent death and pneumonia or improve quality of life, nutritional status, or oral intake in individuals with oropharyngeal dysphagia, which could be attributed to limited numbers of included studies in the review. The mechanisms behind the increased dietary energy intake may include improved bolus cohesion and adhesion, enabling more efficient consumption and larger food portions. The smooth texture and consistency of TMDs require less effort during mastication, which facilitates easier transport and propulsion through the oropharyngeal phases into the esophageal phase and stomach. The smooth texture also enhances sensory feedback, which stimulates saliva production and improves swallowing initiation and bolus formation, and further leads to smoother consumption and potentially larger dietary energy intake. The increased dietary protein intake could be possibly attributed to the fortification of TMDs with protein-rich ingredients and supplements, ensuring sufficient protein to meet nutritional needs. Blending, pureeing, and finely chopping foods in TMDs broke down proteins into smaller particles, and made them easier to swallow.

The meta-analysis also found a non-significant increase in the intake of fat, carbohydrate, sodium, and fiber with TMDs and TFs, which were consistent with previous studies [[20], [21], [22]]. The non-significant increase in fat and sodium might be due to the dietary content of these nutrients remaining unchanged during the processing of TMDs, which could make their intake similar to traditional diets. However, TMDs showed decreases in fiber and carbohydrate intake, possibly due to alterations of the original fiber and carbohydrate contents during processing [58]. Therefore, providing TMDs should be tailored to individual factors such as age, cause and severity of dysphagia, and personal preferences to ensure optimal and adequate dietary nutrient intake while addressing potential nutritional deficiencies.

### 4.2 TFs and aspiration

The current meta-analysis showed that adults with dysphagia who received TFs had a significantly lower risk of aspiration compared to those who received non-TFs. This aligns with a previous study showing reduced incidences of aspiration with TFs in adults with dysphagia [59]. Several factors could explain the reduced risk of aspiration with TFs: (1) TFs increased bolus cohesiveness, hardness, and adhesiveness, creating a more viscous bolus that prevented rapid dispersion and spillage into the trachea, minimizing aspiration risk compared to thin liquids. (2) TFs slowed down fluid flow, which controlled the bolus flow rate, provided more time for the glottis to close, prevented the bolus from entering the trachea, and improved the coordination of swallowing and airway closure. (3) TFs decreased pharyngeal residues by creating a cohesive and adhesive fluid bolus that prevented pooling in the oral cavity and pharynx and reduced aspiration during and after swallowing. (4) Gum-based TFs were resistant to amylase during oral manipulation, helping maintain bolus cohesiveness and adhesiveness, thus preventing oral residues and aspiration. (5) Gum-based TFs maintained stable viscosity over time, preventing fluid residues pooling in the pharynx and reducing aspiration during the pharyngeal phase. The moderator analysis demonstrated that the effectiveness of TFs diminished with an increased age. Older adults often had multiple comorbidities and polypharmacy, which could worsen dysphagia severity. Therefore, dysphagia assessment and management should be included in the routine care of older adults. TFs should be customized based on an individual’s specific swallowing abilities to ensure and promote improved swallowing safety. Continuous assessment is crucial to tailor the management of dysphagia with TFs to an individual’s needs and reduce the risk of aspiration.

### 4.3 TFs and a water protocol on fluid intake

The current meta-analysis revealed that TFs plus a water protocol significantly increased fluid intake compared to TFs alone. However, evidence on the effectiveness of this combined approach remains limited [[20], [21], [22],^60^]. The increased fluid intake could be attributed to several factors. (1) The combination of TFs and a water protocol provided a variety of fluid options, making the drinking experience more enjoyable and encouraging adults with dysphagia to consume more fluids to meet their hydration needs. (2) This approach ensured safer fluid intake without compromising swallowing safety and the hydration status, resulting in a more satisfying and enjoyable drinking experience. (3) The combined protocol promoted oral cleansing and reduced residues by clearing residual food particles and preventing bolus build-up in the mouth and pharynx. Healthcare professionals, including nurses, dietitians, physicians, and speech-language therapists, play a crucial role in encouraging and guiding adults with dysphagia on appropriate fluid intake strategies. Compliance with recommendations and exploring alternative strategies, such as using thickening agents in specific juices that are more palatable, are essential for maintaining and increasing fluid intake in adults with dysphagia. Balancing the need to manage dysphagia-associated complications while maintaining hydration is important for an effective plan to increase fluid intake in adults with dysphagia.

### 4.4 TMDs and body composition

The current meta-analysis found that TMDs had a non-significant small effect on increasing BMI and BW. Evidence on the effectiveness of TMDs on body composition remains limited [[20], [21], [22],^60^]. Several factors might explain the non-significant increase in body composition. (1) Dysphagia persisted among these patients, affecting their intake of dietary nutrients. TMDs were compensatory and did not resolve the underlying dysphagia. (2) The shorter duration of applying TMDs in the included studies could have been insufficient to observe changes in body composition, including BMI and BW. To address these issues, ensuring nutritional adequacy and offering diverse food choices in TMDs are essential to prevent unintentional weight loss that affects BMI. Additionally, TMDs with adequate dietary nutrients might lead to improvement in body composition, including BMI and BW. Early management of dysphagia and individualized dietary approaches with regular monitoring and adjustments are crucial for improving body composition and overall health and well-being of adults with dysphagia.

### 4.5 Strengths and limitations of the study

The current meta-analysis study has several strengths. First, to our knowledge, this meta-analysis extended evidence in the current literature on the effectiveness of TMDs and TFs on dietary nutrient intake (energy, protein, fat, carbohydrate, sodium, and fiber), aspiration, fluid intake, and body composition (BMI and BW). Second, we included a large sample size of 16 RCTs comprising 1,812 adults with dysphagia. Third, we used a rigorous scientific methodology by following PRISMA guidelines, ensuring that all eligible RCTs were included with no restrictions on the year of publication or language. Fourth, we conducted a comprehensive search in Ovid-MEDLINE, Embase, Cochrane Library, PubMed, CINAHL, Web of Science, and reference lists of previously published systematic reviews and meta-analyses, and registered our study protocol with PROSPERO. However, the current meta-analysis has some limitations. First, the included studies had limited follow-up periods, indicating that future RCTs with longer follow-up are needed to determine the short- and long-term benefits of TMDs and TFs. Second, some of the included randomized controlled studies provided limited descriptions of the protocols for TMDs and TFs, making it difficult to assess the dose effect of these interventions. Future rigorous RCTs with detailed protocols for TMDs and TFs are needed to address this issue.

## 5 Conclusions

The current study findings demonstrate that TMDs and TFs increase dietary energy and protein intake among adults with dysphagia. Additionally, TFs are associated with reduced aspiration, while a combination of TFs and a water protocol increase fluid intake in adults with dysphagia. In dysphagia management, TMDs and TFs can serve as compensatory measures to boost dietary nutrient and fluid intake and promote swallowing safety by reducing aspiration. These interventions enhance dietary intake of energy, protein, and fluids, and mitigate the risk of aspiration. Restoring normal swallowing function and preventing dysphagia-related complications remain paramount in managing adults with dysphagia. The high quality of RCTs with larger sample sizes, short-term and long-term follow-up, and adequate details on the TMDs and TFs are needed to further validate the efficacy of these interventions in adults with dysphagia.

## CRediT authorship contribution statement

Y.H.C. and J.C.-J.C. contributed to the conceptualization and methodology. Y.H.C. conducted the study, analyzed the data, and wrote the original manuscript draft. J.C.-J.C. supervised, reviewed, and edited the manuscript. Both authors read and approved the final version of the manuscript.

## Declaration of Generative AI and AI-assisted technologies in the writing process

The authors declare that they have not used generative AI (a type of artificial intelligence technology that can produce various types of content including text, imagery, audio and synthetic data) and AI-assisted technologies in the writing process.

## Funding

This study did not receive any funding.

## Declaration of competing interest

The authors declare no conflict of interests.
