# Supplementary material for: Effectiveness of diet modification on dietary nutrient intake, aspiration, and fluid intake for adults with dysphagia: a meta-analysis of randomized controlled trials
Source: J Nutr Health Aging. 2025 Jan 15;29(4):100486. doi: 10.1016/j.jnha.2025.100486 (PMC12179983; doi:10.1016/j.jnha.2025.100486)
Supplement: Supplementary file 1 [file mmc1.docx]

**Effectiveness of diet modification on dietary nutrient intake, aspiration, and fluid intake for adults with dysphagia: a meta-analysis of randomized controlled trials**

**Supplementary Table S1**

Search strategy.

| Database | Keywords and MeSH terms | Number of studies |
| --- | --- | --- |
| CINAHL | (elderly OR aged OR old age OR adults OR older adults OR old people) AND (modified diet OR modified-texture diet OR puree diet OR pureed diet OR mince diet OR blend diet OR chop diet OR soft diet OR soften diet OR thicken diet OR liquid diet OR liquefied diet OR thickened diet OR fluid thickeners OR thickened fluids OR liquid thickeners OR thickened liquids OR gum-based thickeners OR modified starch thickeners OR starch thickeners) AND (dysphagia OR swallowing disorder OR deglutition disorder OR oropharyngeal dysphagia)  **Expanders** - Apply equivalent subjects  **Search modes** - Find all my search terms | 222 |
| Cochrane Library | elderly OR aged OR old age OR adults OR older adults OR old people in Title Abstract Keyword AND modified diet OR modified-texture diet OR puree diet OR pureed diet OR mince diet OR blend diet OR chop diet OR soft diet OR soften diet OR thicken diet OR liquid diet OR liquefied diet OR thickened diet OR fluid thickeners OR thickened fluids OR liquid thickeners OR thickened liquids OR gum-based thickeners OR modified starch thickeners OR starch thickeners in Title Abstract Keyword AND dysphagia OR swallowing disorder OR deglutition disorder OR oropharyngeal dysphagia in Title Abstract Keyword - (Word variations have been searched) | 174 |
| Embase | ('elderly'/exp OR elderly OR 'aged'/exp OR aged OR 'old age'/exp OR 'old age' OR (old AND ('age'/exp OR age)) OR 'adults'/exp OR adults OR 'older adults'/exp OR 'older adults' OR (older AND ('adults'/exp OR adults)) OR 'old people' OR (old AND people)) AND ('modified diet' OR (modified AND ('diet'/exp OR diet)) OR 'modified-texture diet'/exp OR 'modified-texture diet' OR ('modified texture' AND ('diet'/exp OR diet)) OR 'puree diet'/exp OR 'puree diet' OR (('puree'/exp OR puree) AND ('diet'/exp OR diet)) OR 'pureed diet'/exp OR 'pureed diet' OR (pureed AND ('diet'/exp OR diet)) OR 'mince diet' OR (mince AND ('diet'/exp OR diet)) OR 'blend diet' OR (blend AND ('diet'/exp OR diet)) OR 'chop diet' OR (('chop'/exp OR chop) AND ('diet'/exp OR diet)) OR 'soft diet'/exp OR 'soft diet' OR (soft AND ('diet'/exp OR diet)) OR 'soften diet' OR (soften AND ('diet'/exp OR diet)) OR 'thicken diet' OR (thicken AND ('diet'/exp OR diet)) OR 'liquid diet'/exp OR 'liquid diet' OR (('liquid'/exp OR liquid) AND ('diet'/exp OR diet)) OR 'liquefied diet' OR (liquefied AND ('diet'/exp OR diet)) OR 'thickened diet' OR (thickened AND ('diet'/exp OR diet)) OR 'fluid thickeners' OR (('fluid'/exp OR fluid) AND thickeners) OR 'thickened fluids'/exp OR 'thickened fluids' OR (thickened AND ('fluids'/exp OR fluids)) OR 'liquid thickeners' OR (('liquid'/exp OR liquid) AND thickeners) OR 'thickened liquids'/exp OR 'thickened liquids' OR (thickened AND liquids) OR 'gum-based thickeners' OR ('gum based' AND thickeners) OR 'modified starch thickeners' OR (modified AND ('starch'/exp OR starch) AND thickeners) OR 'starch thickeners' OR (('starch'/exp OR starch) AND thickeners)) AND ('dysphagia'/exp OR dysphagia OR 'swallowing disorder'/exp OR 'swallowing disorder' OR (('swallowing'/exp OR swallowing) AND ('disorder'/exp OR disorder)) OR 'deglutition disorder'/exp OR 'deglutition disorder' OR (('deglutition'/exp OR deglutition) AND ('disorder'/exp OR disorder)) OR 'oropharyngeal dysphagia'/exp OR 'oropharyngeal dysphagia' OR (oropharyngeal AND ('dysphagia'/exp OR dysphagia))) | 1,613 |
| Ovid-MEDLINE | #4. #1 AND #2 AND #3  #3.(dysphagia or swallowing disorder or deglutition disorder or oropharyngeal dysphagia).mp. [mp=title, book title, abstract, original title, name of substance word, subject heading word, floating sub-heading word, keyword heading word, organism supplementary concept word, protocol supplementary concept word, rare disease supplementary concept word, unique identifier, synonyms, population supplementary concept word, anatomy supplementary concept word]  #2.(modified diet or modified-texture diet or puree diet or pureed diet or mince diet or blend diet or chop diet or soft diet or soften diet or thicken diet or liquid diet or liquefied diet or thickened diet or fluid thickeners or thickened fluids or liquid thickeners or thickened liquids or gum-based thickeners or modified starch thickeners or starch thickeners).mp. [mp=title, book title, abstract, original title, name of substance word, subject heading word, floating sub-heading word, keyword heading word, organism supplementary concept word, protocol supplementary concept word, rare disease supplementary concept word, unique identifier, synonyms, population supplementary concept word, anatomy supplementary concept word]  #1.(elderly or aged or old age or adults or older adults or old people).mp. [mp=title, book title, abstract, original title, name of substance word, subject heading word, floating sub-heading word, keyword heading word, organism supplementary concept word, protocol supplementary concept word, rare disease supplementary concept word, unique identifier, synonyms, population supplementary concept word, anatomy supplementary concept word] | 213 |
| PubMed | ("aged"[MeSH Terms] OR "aged"[All Fields] OR "elderly"[All Fields] OR "elderlies"[All Fields] OR "elderly s"[All Fields] OR "elderlys"[All Fields] OR ("aged"[MeSH Terms] OR "aged"[All Fields]) OR ("old"[All Fields] AND ("agrosyst geosci environ"[Journal] OR "age"[Journal] OR "age omaha"[Journal] OR "age dordr"[Journal] OR "adv genet eng"[Journal] OR "age"[All Fields])) OR ("adult"[MeSH Terms] OR "adult"[All Fields] OR "adults"[All Fields] OR "adult s"[All Fields]) OR ("aged"[MeSH Terms] OR "aged"[All Fields] OR ("older"[All Fields] AND "adults"[All Fields]) OR "older adults"[All Fields]) OR ("old"[All Fields] AND ("people s"[All Fields] OR "peopled"[All Fields] OR "peopling"[All Fields] OR "persons"[MeSH Terms] OR "persons"[All Fields] OR "people"[All Fields] OR "peoples"[All Fields]))) AND ((("modifiable"[All Fields] OR "modified"[All Fields] OR "modifier"[All Fields] OR "modifiers"[All Fields] OR "modifies"[All Fields] OR "modify"[All Fields] OR "modifying"[All Fields]) AND ("diet"[MeSH Terms] OR "diet"[All Fields])) OR ("modified-texture"[All Fields] AND ("diet"[MeSH Terms] OR "diet"[All Fields])) OR (("puree"[All Fields] OR "pureed"[All Fields] OR "pureeing"[All Fields] OR "purees"[All Fields]) AND ("diet"[MeSH Terms] OR "diet"[All Fields])) OR (("puree"[All Fields] OR "pureed"[All Fields] OR "pureeing"[All Fields] OR "purees"[All Fields]) AND ("diet"[MeSH Terms] OR "diet"[All Fields])) OR (("mince"[All Fields] OR "minced"[All Fields] OR "minces"[All Fields] OR "mincing"[All Fields]) AND ("diet"[MeSH Terms] OR "diet"[All Fields])) OR (("blend"[All Fields] OR "blend s"[All Fields] OR "blended"[All Fields] OR "blending"[All Fields] OR "blends"[All Fields]) AND ("diet"[MeSH Terms] OR "diet"[All Fields])) OR ("chop"[All Fields] AND ("diet"[MeSH Terms] OR "diet"[All Fields])) OR ("soft"[All Fields] AND ("diet"[MeSH Terms] OR "diet"[All Fields])) OR (("soften"[All Fields] OR "softened"[All Fields] OR "softener"[All Fields] OR "softeners"[All Fields] OR "softening"[All Fields] OR "softenings"[All Fields] OR "softens"[All Fields]) AND ("diet"[MeSH Terms] OR "diet"[All Fields])) OR (("thicken"[All Fields] OR "thickened"[All Fields] OR "thickening"[All Fields] OR "thickenings"[All Fields] OR "thickens"[All Fields]) AND ("diet"[MeSH Terms] OR "diet"[All Fields])) OR (("liquid"[All Fields] OR "liquid s"[All Fields] OR "liquids"[All Fields]) AND ("diet"[MeSH Terms] OR "diet"[All Fields])) OR (("liquefied"[All Fields] OR "liquefies"[All Fields] OR "liquefy"[All Fields] OR "liquefying"[All Fields]) AND ("diet"[MeSH Terms] OR "diet"[All Fields])) OR (("thicken"[All Fields] OR "thickened"[All Fields] OR "thickening"[All Fields] OR "thickenings"[All Fields] OR "thickens"[All Fields]) AND ("diet"[MeSH Terms] OR "diet"[All Fields])) OR (("fluid"[All Fields] OR "fluid s"[All Fields] OR "fluids"[All Fields]) AND ("thickener"[All Fields] OR "thickeners"[All Fields])) OR (("thicken"[All Fields] OR "thickened"[All Fields] OR "thickening"[All Fields] OR "thickenings"[All Fields] OR "thickens"[All Fields]) AND ("fluid"[All Fields] OR "fluid s"[All Fields] OR "fluids"[All Fields])) OR (("liquid"[All Fields] OR "liquid s"[All Fields] OR "liquids"[All Fields]) AND ("thickener"[All Fields] OR "thickeners"[All Fields])) OR (("thicken"[All Fields] OR "thickened"[All Fields] OR "thickening"[All Fields] OR "thickenings"[All Fields] OR "thickens"[All Fields]) AND ("liquid"[All Fields] OR "liquid s"[All Fields] OR "liquids"[All Fields])) OR ("gum-based"[All Fields] AND ("thickener"[All Fields] OR "thickeners"[All Fields])) OR (("modifiable"[All Fields] OR "modified"[All Fields] OR "modifier"[All Fields] OR "modifiers"[All Fields] OR "modifies"[All Fields] OR "modify"[All Fields] OR "modifying"[All Fields]) AND ("starch"[MeSH Terms] OR "starch"[All Fields] OR "starches"[All Fields]) AND ("thickener"[All Fields] OR "thickeners"[All Fields])) OR (("starch"[MeSH Terms] OR "starch"[All Fields] OR "starches"[All Fields]) AND ("thickener"[All Fields] OR "thickeners"[All Fields]))) AND ("deglutition disorders"[MeSH Terms] OR ("deglutition"[All Fields] AND "disorders"[All Fields]) OR "deglutition disorders"[All Fields] OR "dysphagia"[All Fields] OR "dysphagias"[All Fields] OR ("deglutition disorders"[MeSH Terms] OR ("deglutition"[All Fields] AND "disorders"[All Fields]) OR "deglutition disorders"[All Fields] OR ("swallowing"[All Fields] AND "disorder"[All Fields]) OR "swallowing disorder"[All Fields]) OR ("deglutition disorders"[MeSH Terms] OR ("deglutition"[All Fields] AND "disorders"[All Fields]) OR "deglutition disorders"[All Fields] OR ("deglutition"[All Fields] AND "disorder"[All Fields]) OR "deglutition disorder"[All Fields]) OR ("deglutition disorders"[MeSH Terms] OR ("deglutition"[All Fields] AND "disorders"[All Fields]) OR "deglutition disorders"[All Fields] OR ("oropharyngeal"[All Fields] AND "dysphagia"[All Fields]) OR "oropharyngeal dysphagia"[All Fields])) | 670 |
| Web of Science | [**elderly OR aged OR old age OR adults OR older adults OR old people**(All Fields) AND**modified diet OR modified-texture diet OR puree diet OR purged diet OR mince diet OR blend diet OR chop diet OR soft diet OR soften diet OR thicker diet OR liquid diet OR liquefied diet OR thickened diet OR fluid thickeners OR thickened fluids OR liquid thickeners OR thickened liquids OR gum-based thickeners OR modified starch thickeners OR starch thickeners** (All Fields) AND**dysphagia OR swallowing disorder OR deglutition disorder OR oropharyngeal dysphagia**(All Fields)](https://www.webofscience.com/wos/woscc/summary/55dd79a6-415d-43aa-ab03-a127e4098f9a-0135bd9e0b/relevance/1)  https://www.webofscience.com/wos/woscc/summary/cee7bfa3-7eea-4692-b0d1-cf097e5a573c-0135bd9dd1/relevance/1 | 435 |

**Supplementary Table S2**

Results of the moderator analysis for aspiration.

| Variable | *n* | OR (95% CI) | *P* value |
| --- | --- | --- | --- |
| Age | 9 | 0.04 (0.02–0.08) | 0.042 |
| Sample size | 9 | 0.001 (-0.001–0.002) | 0.397 |
| Female, % | 9 | 0.01 (-0.03–0.05) | 0.627 |
| Etiology of dysphagia | 9 | 0.65 (0.52–0.81) | 0.614 |
| Parkinson’s disease | 2 | 0.72 (0.52–0.98) |  |
| Mixed etiology | 5 | 0.59 (0.43–0.81) |  |
| Stroke | 2 | 0.31 (0.02–4.44) |  |
| Type of thickener | 9 | 0.61 (0.46–0.81) | 0.555 |
| Gum-based | 5 | 0.63 (0.47–0.84) |  |
| Starch-based | 4 | 0.48 (0.20–1.12) |  |

CI, confidence interval; *n*, number of studies; OR, odds ratio.

**Supplementary Table S3**

List of excluded studies.

| **Exclusion criteria** | **Authors** | **Study title** |
| --- | --- | --- |
| Similar interventions | Taylor & Barr, 2006 | Provision of small, frequent meals does not improve energy intake of elderly residents with dysphagia who live in an extended-care facility |
|  | Whelan 2001 | Inadequate fluid intakes in dysphagic acute stroke |
| Unavailable full-text | Aktas et al., 2023 | Dietary intakes of individuals with temporomandibular disorders: a comparative study |
|  | Seemer et al., 2022 | Effects of an individualised nutritional intervention to tackle malnutrition in nursing homes: a pre-post study |
| Combined with non-dysphagia patients | Dahl et al., 2005 | Effects of thickened beverages fortified with inulin on beverage acceptance, gastrointestinal function, and bone resorption in institutionalized adults |
| Combined with other dysphagia therapies | Wang et al., 2023 | The therapeutic effect of swallow training with a xanthan gum-based thickener in addition to classical dysphagia therapy in Chinese patients with post-stroke oropharyngeal dysphagia: a randomized controlled study |
| Insufficient outcomes | Bolivar-Prados et al., 2019 | Effect of a gum-based thickener on the safety of swallowing in patients with poststroke oropharyngeal dysphagia |
|  | Dennis 2005 | Routine oral nutritional supplementation for stroke patients in hospital (FOOD): a multicentre randomised controlled trial |
|  | Killeen et al., 2018 | Tolerability and product properties of a gum-containing thickener in patients with dysphagia |
|  | Kyodo et al., 2020 | Pureed diets containing a gelling agent to reduce the risk of aspiration in elderly patients with moderate to severe dysphagia: a randomized, crossover trial |
|  | Sezgin et al., 2018 | The effect of "xanthan gum-based fluid thickener" on hydration, swallowing functions and nutritional status in total maxillectomy patients |


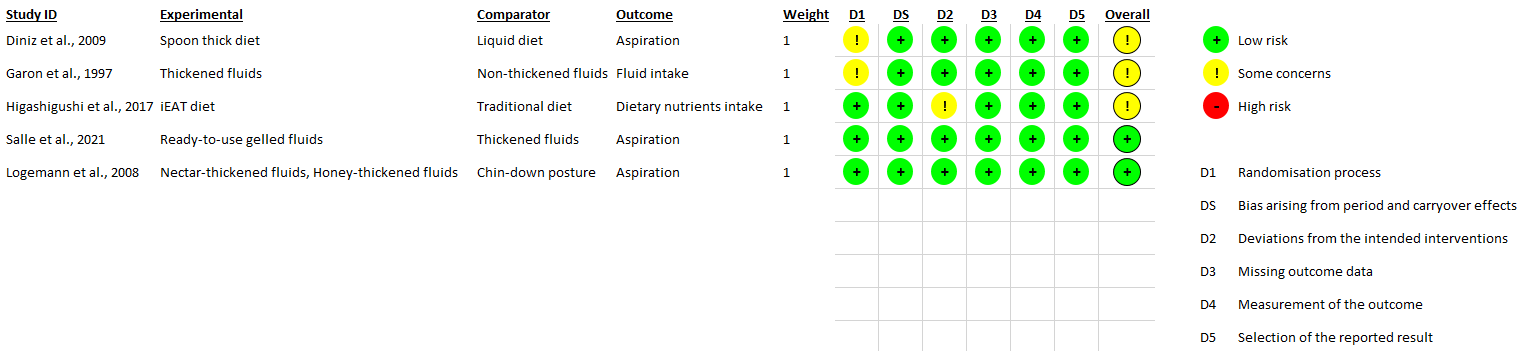


**Supplementary Fig. S1.** Risk of bias assessment for crossover randomized controlled trials.


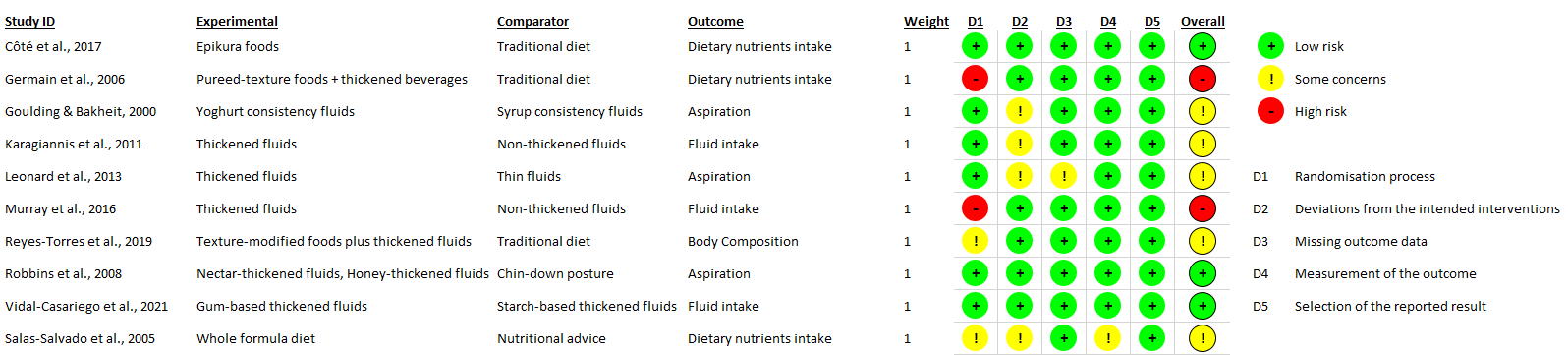


**Supplementary Fig. S2.** Risk of bias assessment for parallel randomized controlled trials.


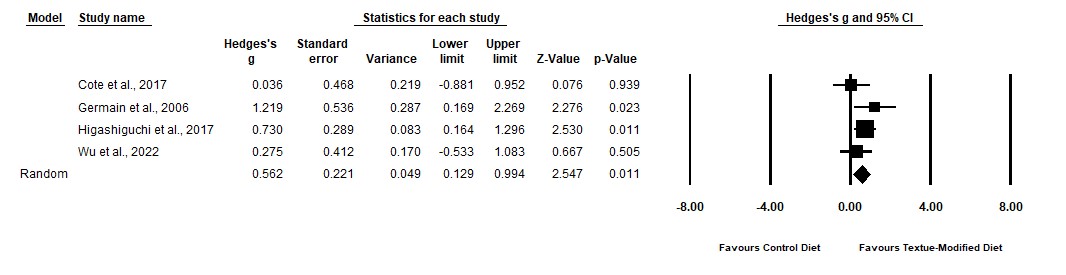


**Supplementary Fig. S3.** Effectiveness of texture-modified diets on protein intake.


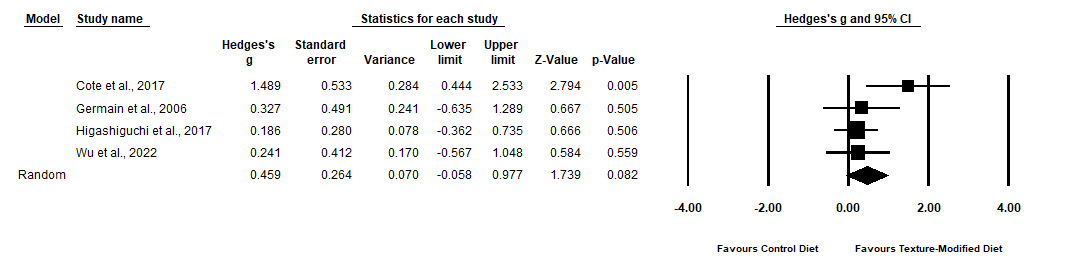


**Supplementary Fig. S4.** Effectiveness of texture-modified diets on fat intake.


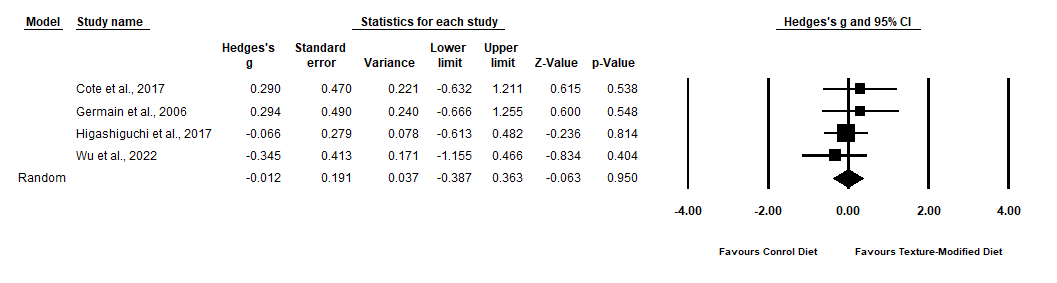


**Supplementary Fig. S5.** Effectiveness of texture-modified diets on carbohydrate intake.


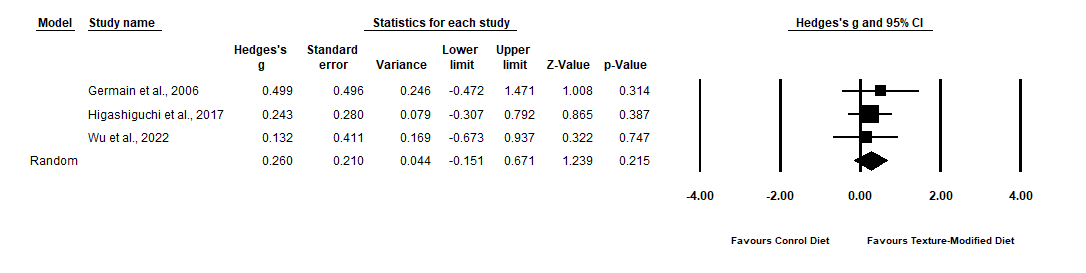


**Supplementary Fig. S6.** Effectiveness of texture-modified diets on sodium intake.


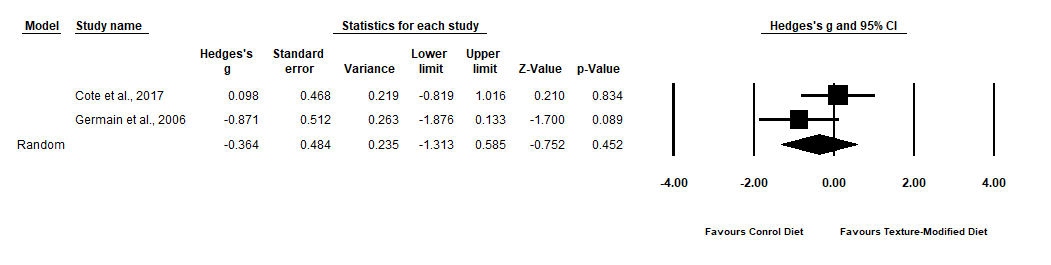
**Supplementary Fig. S7.** Effectiveness of texture-modified diets on fiber intake.


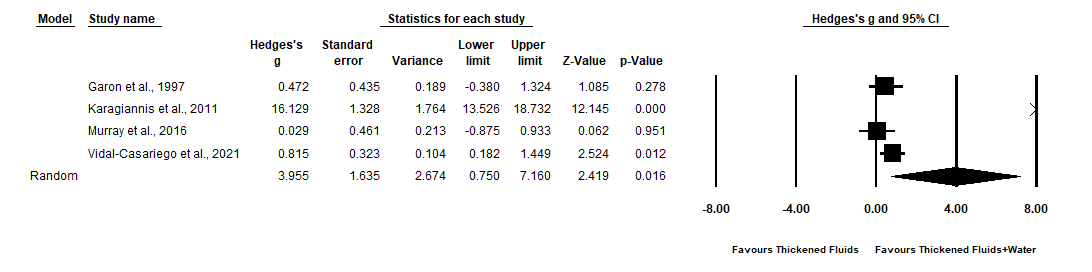


**Supplementary Fig. S8**. Effectiveness of thickened fluids and a water protocol on fluid intake.

**
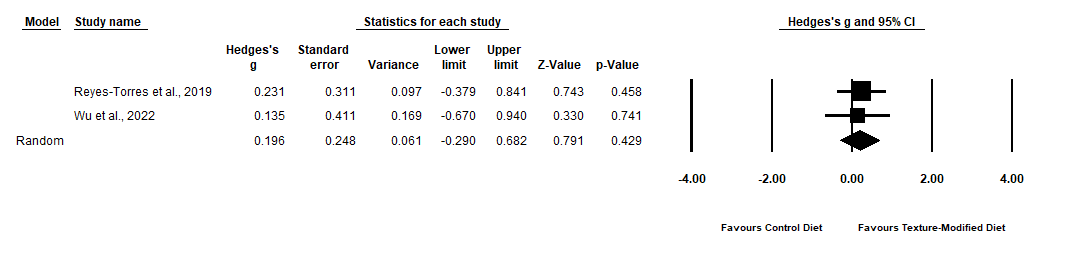
**

**Supplementary Fig. S9.** Effectiveness of texture-modified diets on body mass index.


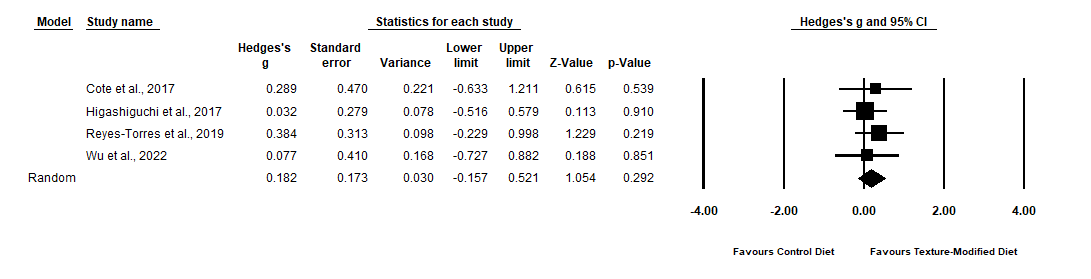


**Supplementary Fig. S10.** Effectiveness of texture-modified diets on body weight.
